# High-Density Electromyography Excitation in Front vs. Back Lat Pull-Down Prime Movers

**DOI:** 10.5114/jhk/185211

**Published:** 2024-04-15

**Authors:** Riccardo Padovan, Nicholas Toninelli, Stefano Longo, Gianpaolo Tornatore, Fabio Esposito, Emiliano Cè, Giuseppe Coratella

**Affiliations:** 1 Department of Biomedical Sciences for Health, Università Degli Studi di Milano, Milan, Italy.

**Keywords:** resistance training, strength training, muscle, eccentric

## Abstract

The current study compared the spatial excitation of the primary muscles during the lat pull-down exercise with the bar passing in front (front-LPD) or behind the neck (back-LPD) using high-density electromyography. Fourteen resistance trained men performed a front-LPD or a back-LPD within a non-fatiguing set with 8-RM as the external load. The muscle excitation centroid of latissimus dorsi, middle trapezius, pectoralis major, biceps brachii, triceps brachii and posterior deltoid muscles were recorded during the ascending and the descending phase. During the descending phase, the front-LPD showed superior excitation of the latissimus dorsi (ES = 0.97) and the pectoralis major (ES = 1.17), while in the ascending phase, the back-LPD exhibited superior excitation of the latissimus dorsi (ES = 0.63), and the front-LPD showed superior excitation of the biceps brachii (ES = 0.41) and the posterior deltoid (ES = 1.77). During the descending phase, the front-LPD showed a more lateral centroid of the latissimus dorsi (ES = 0.60), the biceps brachii (ES = 0.63) and the triceps brachii (ES = 0.98), while the centroid was more medial for the middle trapezius (ES = 0.58). The centroid of the middle trapezius was also more medial in the front-LPD during the ascending phase (ES = 0.85). The pectoralis major centroid was more cranial in the front-LPD for both the descending (ES = 1.58) and the ascending phase (ES = 0.88). The front-LPD appears to provide overall greater excitation in the prime movers. However, distinct spatial excitation patterns were observed, making exercise suitable for the training routine.

## Introduction

Resistance training is based on various exercises that target specific muscle groups to induce adaptations in strength and muscular size. Each exercise has its own biomechanical characteristics that determine the amount of mechanical stimuli each muscle group undergoes (Coratella, 2022). However, considering the possible variations for a given exercise may be useful to target the prime movers differently, depending on the variation executed. The main way to quantify the activity of each muscle group during different variations of the same exercise is the assessment of the electromyographic (EMG) signal, which measures the single muscle excitation during a given movement (Vieira and Botter, 2021). The literature has therefore described how the main prime movers excite during different variations of many exercises, such as the bench press (Cabral et al., 2022; Coratella et al., 2020b; Jaworski et al., 2020; Rawska et al., 2019; Saeterbakken et al., 2021; Stastny et al., 2017; Strońska et al., 2018; Tsoukos and Bogdanis, 2023; Wojdala et al., 2022), the squat (Clark et al., 2012; Coratella et al., 2021; van den Tillaar et al., 2019), the deadlift (Andersen et al., 2019; Coratella et al., 2022a; Martín-Fuentes et al., 2020), the overhead press (Błażkiewicz and Hadamus, 2022; Coratella et al., 2022b; Stronska et al., 2018), the biceps curl (Coratella et al., 2023a, 2023b; Marcolin et al., 2018), the rower (Fujita et al., 2020), the lateral raise (Coratella et al., 2020a; Reinold et al., 2007), and the lat pull-down (LPD) (Andersen et al., 2014; Signorile et al., 2002; Sperandei et al., 2009). Particularly, the LPD is used to stimulate the upper body muscles with a specific focus on the torso, i.e., the latissimus dorsi, the trapezius, the pectoralis major, the biceps brachii, the triceps brachii and the posterior deltoid, although, previous studies have not examined them all together, thus resulting in a partial view of the potential muscular benefits deriving from the incorporation of the LPD in the training routine.

Given the numerous variations possible for performing the LPD, previous literature has examined the effects of grip distance (Andersen et al., 2014) and of the bar trajectory, i.e., in front (front-LPD) or behind the head (back-LPD) (Signorile et al., 2002; Sperandei et al., 2009). The main results are that both the wide and the narrow grip induced similar excitation in the latissimus dorsi, the middle trapezius and the biceps brachii (Andersen et al., 2014). Moreover, the front- and the back-LPD differ in terms of muscle excitation, with the front-LPD inducing greater excitation in the pectoralis major (Sperandei et al., 2009), the latissimus dorsi, the triceps brachii and the posterior deltoid (Signorile et al., 2002) while the back-LPD shows higher excitation for the posterior deltoid and the biceps brachii (Sperandei et al., 2009).

However, previous results offer only a quantitative analysis of muscle excitation. In recent years, the advent of high-density EMG has provided a deeper understanding of the role each muscle has during a single movement (Vieira and Botter, 2021). Such a technology allows for a spatial localization of the EMG signal by means of the grid instead of a single pair of electrodes (Vieira and Botter, 2021). This permits to identify to what extent fascicles are involved during a given movement (Vieira and Botter, 2021), hence going through the architectural complexity of most muscles. For example, this approach has been used for examining different exercises targeting hamstrings (Hegyi et al., 2019), or to investigate how the pectoralis major fascicles excite during the flat vs. the inclined bench press (Cabral et al., 2022).

When investigating a dynamic resistance exercise, the ascending and the descending phase often correspond to the concentric and eccentric contraction of the prime movers. Given the acute (Duchateau and Enoka, 2016), short-term (Coratella and Bertinato, 2015) and long-term concentric vs. eccentric differences (Coratella et al., 2022c, 2022d), a separate examination of muscle excitation during the two phases is warranted. Therefore, the present study aimed to compare the spatial excitation of the primary muscles during the front- vs. the back-LPD, separating the descending (mostly concentric actions) from the ascending phase (mostly eccentric actions).

## Methods

### Participants

Based on the effect size calculated from the comparison of the excitation for the latissimus dorsi in the front- vs. the back-LPD recorded in a previous study (Signorile et al., 2002), an *a priori* sample size was determined using G*Power for a two-way repeated-measures analysis of variance (ANOVA-RM) with α = 0.05, power = 0.95 and ES = 2.26, resulting in 12 subjects. To prevent the effects of possible dropouts, 14 resistance trained male participants (age: 23.93 ± 4.14 yrs; stature: 1.78 ± 0.05 m; body mass: 78.08 ± 6.79 kg) were included. All participants had at least three-year experience in resistance training. To be included in the current study, they had to be free from any musculoskeletal injuries in the upper limbs, the glenohumeral joint, and the spine in the last six months and were asked to abstain from alcohol, caffeine, and similar substances in the 24 h preceding the test. This project was approved by the Ethics Committee of the University of Milan (approval code: CE 27/17; approval date: 10 March 2017) and carried out following the Declaration of Helsinki (1964 and updates) for studies involving human subjects. After a full explanation of the purposes of the study and the experimental procedures, participants signed a written informed consent form, and they were informed to be free to withdraw at any time.

### Design and Procedures

The present investigation was designed as a cross-over, repeated measures, within-subject study and was conducted in agreement with previously described procedures (Coratella et al., 2023a, 2023b). Participants were involved in three different sessions. In the first session participants became familiar with the exercise technique defined for the front- and the back-LPD, and we identified the best electrodes’ position for each muscle. During the second session, the 8-repetition maximum (8-RM) (Coratella et al., 2023b) was determined for the front- and the back-LPD in random order. The third session was initially focused on identifying the maximum voluntary isometric excitation of the examined muscles and, after at least 10 min of passive recovery, EMG data collection was carried out during a non-exhaustive set for the front- and the back-LPD technique. The load was based on the 8-RM and four repetitions were performed to avoid fatigue. A minimum of three days separated each session. Participants were also instructed to abstain from any additional forms of resistance training during the study period.

**Figure 1:**
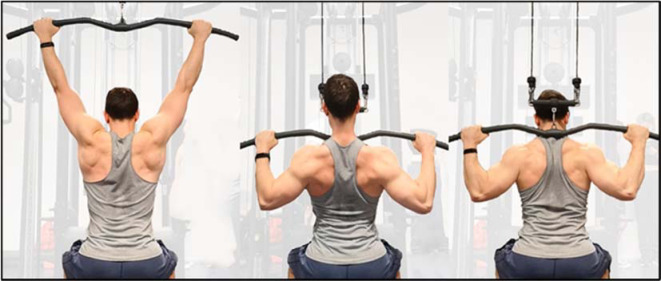
Presentation of the lat pull-down technique. From the left: the starting position, the end of the front and the back variation.

The front-LPD and the back-LPD were performed with a Selection 700 lat pull-down [Technogym, Gambettola (FC), Italy]. The technique of both exercises is illustrated in Figure 1. For both the front- and the back-LPD, in the starting position the hands were prone, the elbows extended, the thighs blocked by the padding to keep the pelvis attached to the seat throughout the whole movement. The hands distance was 1.5 of the inter-acromion distance in both exercises. The descending phase consisted of pulling the bar to the middle chest passing in front of the head for the front-LPD, and to the lowest point of the neck behind the head for the back-LPD. The trunk was allowed to slightly extend and flex, respectively, and an operator visually checked for avoiding any enhancement in the trunk movement. In both the front- and the back-LPD, participants were instructed to adduct the arms on a lateral plane, minimizing the arm flexion/extension on the sagittal plane. Once completed the descending phase, an isometric stop of 0.5 s was given and then participants performed the ascending phase, ending with the bar in the highest position. As such, following the appropriate description of resistance exercise technique (Coratella, 2022), a full range of movement was achieved, with the descending and the ascending phase lasting 2 s each as punctuated by a metronome, and with an external focus.

The 8-RM was evaluated using the same exercise technique explained previously in accordance with established protocols (Coratella et al., 2023a, 2023b). Briefly, after performing a standardized warm-up involving 3 sets of 15 repetitions of the LPD with increasing self-selected loads, the 8-RM was determined by progressively increasing the load until participants were unable to complete the descending phase after the 8^th^ repetition in accordance with the technique previously described, indicating failure (Kompf and Arandjelović, 2016). Each attempt was separated by a minimum of three minutes of passive recovery, and participants were strongly encouraged to perform each trial to their best in accordance with standardized instructions. The procedure was repeated in the front-LPD and the back-LPD in random order.

The EMG signal was recorded using semi-disposable high-density electrodes arranged in a grid of 13 rows of 5 columns (GR08MM1305 model, inter-electrode distance of 8 mm, OT Bioelettronica Turin, Italy) while performing the front- and back-LPD sets. The grids were selected following the consensus for the experimental design in electromyography instructions (Besomi et al., 2019). The muscles investigated were the latissimus dorsi, the middle trapezius, the pectoralis major, the biceps brachii, the triceps brachii and the posterior deltoid on the dominant side. The grid in the latissimus dorsi, the biceps brachii, the triceps brachii and the posterior deltoid was placed parallel to the muscle fibers, i.e., longitudinally (Barbero et al., 2012). The grid in the middle trapezius and the pectoralis major was placed perpendicularly to the muscle fibers (Vieira and Botter, 2021). The innervation zone was avoided for latissimus dorsi, middle trapezius and pectoralis major muscles in accordance with the Atlas of Muscle Innervation Zones (Barbero et al., 2012), while the grid size covered the innervation zone in biceps brachii, triceps brachii and posterior deltoid muscles as in previous studies (Campanini et al., 2022; Merletti and Muceli, 2019; Rodriguez-Falces et al., 2013). For these three muscles, the grid was positioned avoiding the cross-talk from adjacent muscles (Vieira and Botter, 2021). For the latissimus dorsi, the grid was placed approximately two fingerbreadths below the lower margin of the humerus (Barbero et al., 2012). EMG of the middle trapezius was recorded by applying the electrode grid on the upper part, approximately 1/3 of the distance between the spinous process of the seventh cervical vertebrae and the acromial angle (Barbero et al., 2012). For the pectoralis major, the grid was positioned on the sternocostal portion, approximately 2 cm away from the lateral margin of the sternum (Barbero et al., 2012). For the biceps brachii, the grid was positioned on the line between the medial acromion and the fossa cubit at the 2/3 cranial portion (Barbero et al., 2012). For the triceps brachii, the grid was placed on the muscle belly, approximately 1/3 of the distance between the acromion and the medial epicondyle of the humerus (Barbero et al., 2012). For the posterior deltoid, the grid was placed on the upper part, approximately 2 cm away from the lateral edge of the acromion (Barbero et al., 2012). The cavities of the electrode grid were completely filled with conductive cream (ac cream, Spes Medica s.r.l. Genoa, Italy) to assure proper electrode skin contact. Prior to attaching the electrode grid, the skin was shaved, abraded and cleaned with alcohol. The EMG signal was recorded in monopolar configuration at 2048 Hz, amplified, and band-pass filtered (20–400 Hz) by an electromyographic system (EMG-USB2+, OT Bioelettronica Turin, Italy) (Merletti et al., 2001a). The reference electrode for monopolar acquisition was placed on the wrist.

Once the participants were equipped with grids, the maximum voluntary isometric excitation was performed for each muscle examined according with the Atlas of Muscle Innervation Zones (Barbero et al., 2012). For the latissimus dorsi, participants were seated with the arm along the torso and the forearm flexed at 90°. From this position, they performed an isometric extension of the humerus against resistance, associated with a depression of the shoulders (Barbero et al., 2012). For the trapezius, participants were seated on a chair, grabbing the handrests and performing the adduction of the shoulder blades (Barbero et al., 2012). For the pectoralis major, participants were standing with one arm abducted at about 30° and they performed an isometric adduction of the arm against an immovable resistance (Barbero et al., 2012). For the biceps brachii, participants were instructed to flex the elbow with the hand supinated against manual resistance, holding the body and the arm attached to the torso (Barbero et al., 2012). For the triceps brachii, participants were standing with one arm close to the body and performing an isometric extension of the elbow at 45° (Barbero et al., 2012). For the posterior deltoid, participants were instructed to produce maximal force during retropulsion of the arm abducted at 90° against resistance applied at the elbow level (Barbero et al., 2012). Three attempts lasting 5 s were performed to determine the individual maximum muscle excitation. A recovery of 3 min separated each attempt (Coratella et al., 2023b). During each attempt the operators provided strong, standardized verbal encouragement to ensure maximum effort from participants. After a passive recovery of 10 minutes, a non-exhaustive set was performed for the front- and the back-LPD, in randomized order. The recovery between the two series was 3 minutes and the load was the 8-RM previously established. In these series four repetitions were performed, to avoid fatigue and to avoid the decay of the execution technique. The cadence of 2 s for the descending and ascending phases was marked by an operator, making sure that there were no accelerations or slowdowns during the movement.

The detection of EMG by electrode grids allows to determine the spatial distribution of muscle excitation beneath the grid. To characterize the spatial distribution, the barycenter in RMS values in vertical and horizontal axis (expressed as mm along the y-axis and x-axis relative to the grid) was calculated and defined as central locus activation (Watanabe et al., 2012). The centroid was defined as the barycenter (Gallina et al., 2013) of the EMG amplitude values along the rows and columns of the electrode grids and determined for latissimus dorsi, middle trapezius, pectoralis major, biceps brachii, triceps brachii, and posterior deltoid muscles.

Surface electromyography (EMG) was obtained in monopolar derivation and then converted to a digital format at a rate of 2048 Hz using a 12-bit analog-to-digital converter with a dynamic range of 5 volts. Prior to digitization, EMG underwent amplification with a variable factor, employing a 20–400-Hz bandwidth amplifier (EMG-USB2, OT Bioelettronica, Turin, Italy) and a CMRR >100 dB (Merletti et al., 2001). EMG signals were characterized in the time domain by the RMS. A 1-s interval was analysed for the maximum voluntary isometric excitation whereas a 250-ms window was used to compute the RMS during the ascending and descending phases of each exercise. During each exercise, the RMS was calculated and averaged on the second central of both the ascending and descending phases. A digital camera attached to a tripod (Iphone 12, 12 MP resolution, 1080 p format, 60 fps, Apple, California) was used to synchronize EMG with the duration of each phase, which was then used to mark the start and the end of each phase while analyzing the EMG signal (Cabral et al., 2022). EMG data were averaged after excluding the first repetition of each set to ensure consistent technique (Marri and Swaminathan, 2016). Subsequently, the EMG RMS of each muscle during each exercise was normalized to its respective maximum voluntary isometric excitation, according to the consensus for the experimental design in electromyography projects (Besomi et al., 2020).

**Figure 2:**
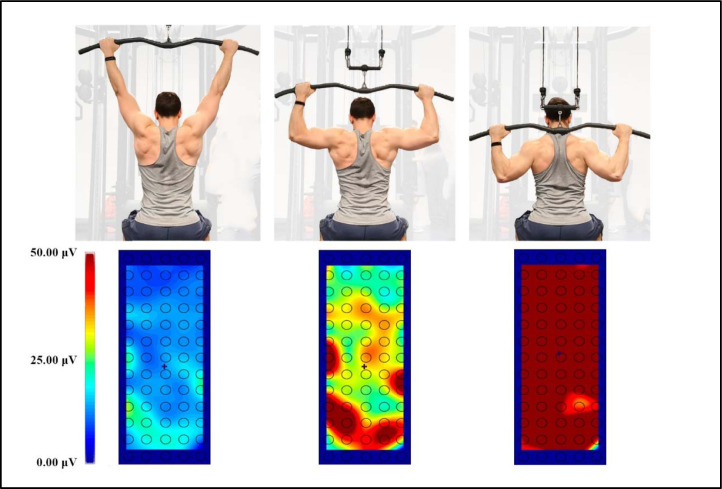
A typical spatial map of muscle excitation for the latissimus dorsi during the back lat pull-down. The panel shows three distinct positions, and the corresponding spatial excitation is reported below each position. The centroid is represented by “+”.

A color map of muscle excitation was obtained upon the RMS of all 64 channels of the grid (Figure 2), using the OTBiolab+ software (v1.9.5.3, OT Bioelettronica, Turin, Italy). To delineate the spatial distribution pattern, the centroid of muscle activation was determined. The spatial maps were estimated by software interpolating the Average Rectified Values (ARV) obtained from the EMG amplitudes on the different electrodes. The barycenter was estimated as the point, along both the electrode grid rows and columns, which balanced the ARV values weighted by their distance from the barycenter. The location of the centroid was defined translating the position of each electrode in the matrix in x- and y-coordinates, expressed in millimetres along the two axes within 250-ms time-windows.

### Statistical Analysis

Statistical analysis was carried out using SPSS 28.0 software (IBM, Armonk, NY, USA). The normality of the data was assessed by conducting the Shapiro-Wilk test and all distributions were found to be normal. Descriptive statistics were reported for a sample size of 14 participants as mean (SD). A two-way ANOVA for repeated measures was used to calculate the front- vs. the back-LPD differences in nRMS and in centroids during the ascending and the descending phase for each muscle. Multiple comparisons were adjusted using Bonferroni's correction, and the mean difference with the 95% confidence interval (95% CI) was reported. The level of significance was set at α < 0.05. The magnitude of interactions and main effects were calculated using partial eta squared (&nbsp;ηp^2^) and interpreted as trivial (up to 0.009), small (0.010 to 0.059), medium (0.060 to 0.139), and large (≥0.140) (Cohen, 1988). Pairwise comparisons were reported as the mean with a 95% confidence interval with Cohen's *d* effect size (ES). The effect size was interpreted according to Hopkins' recommendations: trivial (0.00–0.19), small (0.20–0.59), moderate (0.60–1.19), large (1.20–1.99), and very large (≥2.00) (Hopkins et al., 2009).

## Results

The average 8-RM load was 70.0 (11.6) kg for the front-LPD and 59.3 (10.5) kg for the back-LPD (*p* < 0.01; ES = 1.01, 0.35 to 1.62).

**Figure 3:**
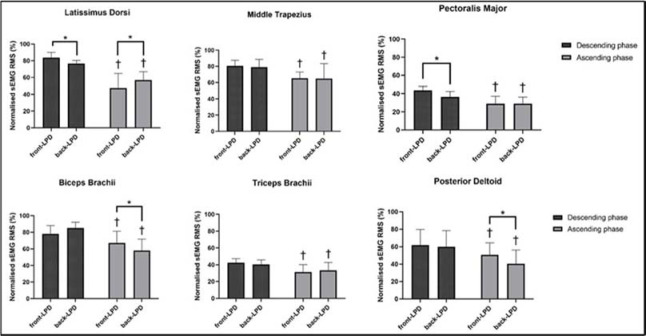
The mean (SD) of the normalized root mean square (nRMS) recorded during the ascending and the descending phase of the front (front-LPD) and the back lat pull-down (back-LPD) is shown for each muscle. Besides the front vs. back lat pull-down differences, nRMS was greater during the descending than the ascending phase in all exercises. *: p < 0.05 vs. back-LPD. †: p < 0.05 vs. the descending phase.

Figure 3 shows the nRMS recorded in all muscles during the descending and the ascending phase of the front- and the back-LPD. An exercise x phase interaction was observed for the nRMS in latissimus dorsi (F = 14.230, *p* < 0.001, ηp2 = 0.354), pectoralis major (F = 5.253, *p* = 0.030, ηp2 = 0.168), biceps brachii (F = 6.533, *p* = 0.017, ηp2 = 0.201), and posterior deltoid (F = 8.647, *p* = 0.007, ηp2 = 0.250) muscles, while no interaction was observed in the middle trapezius (F = 0.018, *p* = 0.894, ηp2 = 0.001) and the triceps brachii (F = 1.825, *p* = 0.188, ηp2 = 0.066). During the descending phase, nRMS was greater in the front- than the back-LPD in the latissimus dorsi (7.21%, 0.91% to 13.52%; ES = 0.97, 0.32 to 1.60) and the pectoralis major (7.30%, 2.56% to 12.04%; ES = 1.17, 0.47 to 1.84), while middle trapezius, biceps brachii, posterior deltoid and triceps brachii muscles had similar excitation (*p* > 0.05). During the ascending phase, nRMS was greater in the back- vs. the front-LPD in the latissimus dorsi (9.14%, 2.84% to 15.44%, ES = 0.63, 0.05 to 1.20), while greater in the front-LPD in the biceps brachii (9.28%, 0.19% to 18.38%, ES = 0.41, −0.14 to 0.95) and the posterior deltoid (10.29%, 5.98% to 14.59%, ES = 1.77, 0.90 to 2.60). No difference was recorded in middle trapezius, pectoralis major and triceps brachii muscles. The descending phase exhibited greater nRMS compared to the ascending phase in all muscles during both the front- and the back-LPD (*p* < 0.05 for all comparisons).

**Figure 4:**
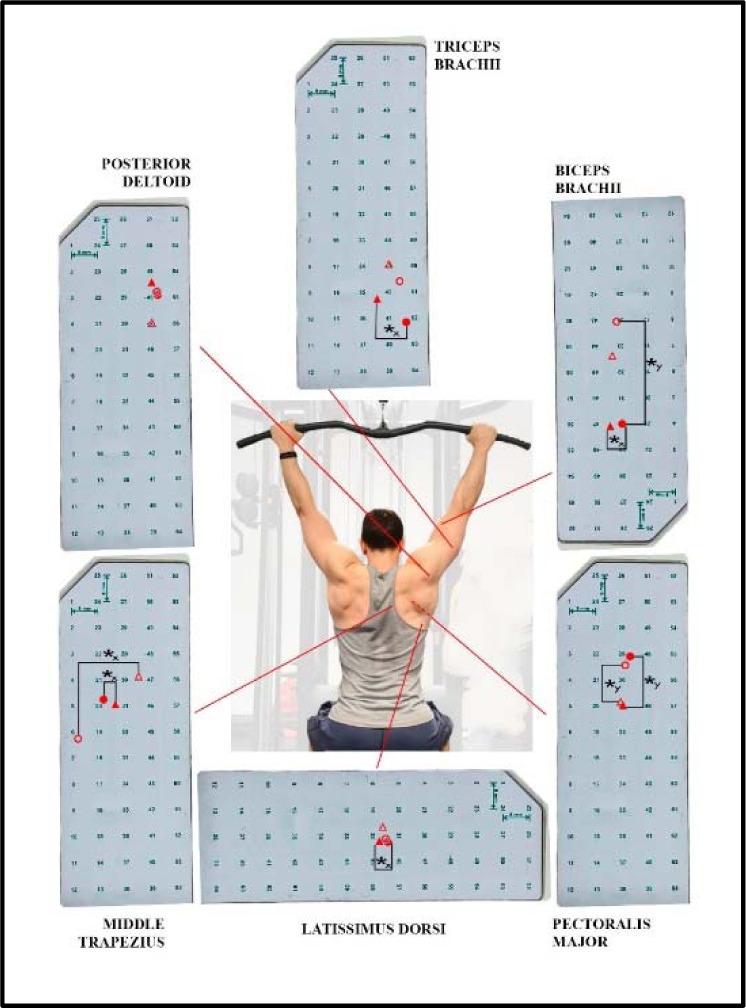
Presentation of the spatial muscle activation for the muscles analyzed. The grids are visualized as positioned on each muscle. The upward and downward directions indicate a cranial and caudal shift in the vertical plane, respectively; the rightward and leftward shifts indicate a lateral and medial shift in the horizontal plane, respectively. The front lat pull-down (front-LPD) is represented graphically by filled circles (⚫) for the descending and empty circles (◉) for the ascending phase. The back lat pull-down (back-LPD) is represented graphically by filled triangles (▲) for the descending and empty triangles (△) for the ascending phase. ✱y: p < 0.05 comparing the centroid on the vertical y-axis ✱x: p < 0.05 comparing the centroid on the horizontal x-axis

Figure 4 displays the average horizontal and vertical coordinates of the centroid for each individual muscle during the descending and ascending phases of both the front- and the back-LPD. No exercise x phase interaction was observed for the horizontal axis in latissimus dorsi (F = 0.540, *p* = 0.469, ηp2 = 0.022), middle trapezius (F = 0.375, *p* = 0.546, ηp2 = 0.015), pectoralis major (F = 0.210, *p* = 0.651, ηp2 = 0.009), biceps brachii (F = 1.143, *p* = 0.296, ηp2 = 0.045), triceps brachii (F = 2.667, *p* = 0.116, ηp2 = 0.100) and posterior deltoid (F = 0.010, *p* = 0.923, ηp2 = 0.000) muscles.

During the descending phase, the centroid was more lateral in the front- vs. the back-LPD in latissimus dorsi (0.21%, 0.01% to 0.41%; ES = 0.60, 0.00 to 1.18) and triceps brachii (−0.87%, −1.36% to −0.38%, ES = 0.98, −1.52 to −0.28) muscles, while the middle trapezius (−0.55%, −1.07% to −0.02%; ES = 0.58, −1.16 to 0.02) and the biceps brachii (−0.37%, −0.68% to −0.06%, ES = 0.63, −1.22 to −0.02) showed an opposite behaviour with the centroid placed more medially. No lateral difference was observed in the centroid in pectoralis major and posterior deltoid muscles (*p* > 0.05). During the ascending phase, the centroid was more medial in the middle trapezius during the front- vs. the back-LPD (−0.77%, −1.29% to −0.24%; ES = 0.85, −1.48 to −0.20), with no further differences in latissimus dorsi, pectoralis major, biceps brachii, triceps brachii and posterior deltoid muscles (*p* > 0.05). No between-phase difference was observed in any muscle (*p* > 0.05). No exercise x phase interaction was observed in the vertical axis for latissimus dorsi (F = 0.876, *p* = 0.359, ηp2 = 0.035), middle trapezius (F = 2.180, *p* = 0.153, ηp2 = 0.083), pectoralis major (F = 0.644, *p* = 0.430, ηp2 = 0.026), biceps brachii (F = 1.492, *p* = 0.234, ηp2 = 0.059), triceps brachii (F = 0.059, *p* = 0.809, ηp2 = 0.002), and posterior deltoid (F = 2.735, *p* = 0.111, ηp2 = 0.102) muscles. The centroid was located more cranially in the front- vs. the back-LPD in the pectoralis major during the descending (1.51%, 0.86% to 2.17%; ES = 1.58, 0.74 to 2.40) and the ascending phase (1.15%, 0.49% to 1.81%; ES = 0.88, 0.22 to 1.51), with no further between-exercise difference (*p* > 0.05). The centroid was more caudal in the biceps brachii during the descending vs. the ascending phase of the front-LPD (−3.05%, −5.76% to −0.34%, ES = 0.91, −1.55 to 0.25). No further between-phase difference was found (*p* > 0.05).

## Discussion

The current study aimed to investigate whether different LPD variations would influence the excitation of the primary muscles during the descending and the ascending phase, using for the first time the high-density EMG. The main results were that: i) the front-LPD showed greater external loads than the back-LPD; ii) during the descending phase, latissimus dorsi and pectoralis major muscles exhibited higher excitation in the front-LPD and during the descending phase the latissimus dorsi showed greater excitation in the back-LPD, while biceps brachii and posterior deltoid muscles showed higher excitation in the front-LPD; iii) in the horizontal plane, the front LPD induced a lateral shift in the centroid during the descending phase for latissimus dorsi and triceps brachii muscles and a medial shift for the biceps brachii, while the medial shift was induced for the middle trapezius during both phases; iv) on the vertical plane, the front-LPD induced a cranial shift in the centroid for the pectoralis major during both phases. Besides the amplitude of the prime movers’ excitation overall favourable in the front-LPD, the analysis of the centroid offers a deeper understanding of both LPD techniques.

Although the relative external load was similar, the absolute external load was overall greater when performing the front- than the back-LPD. This is a preliminary consideration since the load itself may affect muscle excitation. The biomechanical aspects of a given exercise variation may advantage or disadvantage the ability to lift greater loads (Coratella et al., 2021), and this should be acknowledged when examining the role of each prime mover. The present outcomes differ from the only previous study that reported the external load when comparing the front- vs. the back-LPD, which reported no difference in the absolute external load used (10-RM) (Signorile et al., 2002), while another study used the same absolute external load for both variations (Sperandei et al., 2009). The difference between the previous and the current results could be explained by the different resistance training experience of participants, since the present resistance trained men would have developed a greater differentiation between the two techniques and the current training routine as previously reported (Stronska et al., 2022). The greater external load lifted in the front- than the back-LPD is indeed quite common in the gym environment.

The use of high-density EMG allows for a larger muscle area to be examined compared to the pair of single electrodes (Vieira and Botter, 2021). Consequently, comparing the present results with the data reported in the literature needs consider this crucial aspect, since this is the first study where high-density EMG has been used for this purpose. Moreover, and in addition to the greater load used for the front-LPD as mentioned above, some important kinematic considerations should be made. First, while sharing the same starting point, we speculate that the distance covered by the external load is greater during the front- than the back-LPD as required by the technique, leading to greater movement velocity for the same tempo. The nRMS amplitude is known to be greater when faster movements are exerted (Frost et al., 2008), thus this should also be considered in the comparison. That said, during the descending phase, Signorile et al. (2002) found that the front-LPD exhibited higher muscle excitation in the latissimus dorsi, although in the study by Sperandei et al. (2009), no such a difference was found. Similarly, pectorals major muscle was more excited in the front- than in the back-LPD, which is in line with Sperandei et al. (2009), yet in contrast with Signorile et al. (2002) where no difference was found. Both muscles are strong arm adductors in the frontal plane, and while the greater involvement of the pectoralis major is thought due to a more anterior plane of movement, the latissimus dorsi may be expected to act more in the back-LPD considering the more posterior trajectory of the external load found in a previous anterior vs. posterior comparison in the overhead press exercise (Coratella et al., 2022b). The concerns raised above, especially the greater absolute external load for the front-LPD, may help explain these results. Interestingly, the neural pattern is reversed during the ascending phase for the latissimus dorsi which is more excited during the back-LPD. It is possible that more control is needed when starting the movement behind the neck, as it is a less comfortable position in terms of individual capacity. Middle trapezius muscle plays an important role in adducting the scapulae during both the front- and the back-LPD, and the lack of difference observed here highlights that both movements require similar high-level excitation. The literature offers no comparison, thus the present study provides novel outcomes. As for biceps brachii, triceps brachii and posterior deltoid muscles, the two variations recruit them similarly during the descending phase. However, biceps brachii and posterior deltoid muscles are more excited in the front- than the back-LPD during the ascending phase. Considering the bicep brachii, the only previous available data reported no difference during the ascending phase, and more excitation in the back-LPD during the descending phase (Sperandei et al., 2009). Explaining possible muscular differences beyond the methodological aspects is challenging, thus we prefer avoiding further speculations. Regarding the posterior deltoid, the present outcomes are in contrast to previous studies showing that during the descending phase, the posterior deltoid was more excited in the backward (Sperandei et al., 2009) and frontward variations (Signorile et al., 2002). On the one hand, the posterior deltoid is expected to control for the posterior trajectory of the bar, as seen when pushing behind the neck (Coratella et al., 2022b). Yet, posterior deltoid is more elongated when acting frontward, and this results in greater excitation (Coratella et al., 2020a). It is possible that the second condition prevailed here.

The major novel outcomes concern the spatial excitation within each muscle. The more medial placement of the centroid during the front-LPD may depend on the greater scapular adduction required to end the movement in a more caudal position, possibly involving more medial fascicles. This is accompanied by a small lateral shift, visible only during the descending phase, in the centroid of the latissimus dorsi. Possibly, the inter-play between the scapular and the arm adduction may demand a more pronounced action of the former during the front-LPD to bring the bar to the chest, and vice versa for the back-LPD. Moreover, the frontal trajectory of the bar may have elongated the fascicle of the latissimus dorsi involved in the exercise, resulting in a more lateral recruitment. The pectoralis major had a cranial shift of the centroid during the front-LPD. It should be noted that the grid was placed on the central portion of the pectoralis major, so that the upper fascicles may have been more involved in the anterior control of the external load, as previously shown (Coratella et al., 2020a, 2022b). The biceps brachii had its centroid shifted medially in the front- compared to the back-LPD. It is possible that the frontward variation allows for a greater forearm flexion, and the short head of the biceps, more medial than the long head, may have been slightly more involved. Interestingly, the biceps brachii appeared to shift cranially the centroid during both the descending and the ascending phase, albeit the former was just a trend. Although speculative, it is possible that releasing the external load from the lowest point may have required greater control of the arm than the forearm flexion, and this may have involved more the cranial fascicles of the biceps brachii. The centroid of the triceps brachii was more lateral during the front- LPD. During the back-LPD the arm ends in a more extended position in the sagittal plane compared to the front-LPD. The long head of the triceps brachii is the only head responsible for that between the three, and is placed more medially than the single-joint lateral and medial heads. Thus, its greater involvement is possible, making the overall centroid more medial. Lastly, no difference in the centroid was observed in the posterior deltoid, implying that the demands of its actions are possibly similar.

The main limitation of the present study is that the outcomes are referred to the combination of the technique as described, the external load and the background of the participants. Changing one or more of the three may impact the results. Consequently, the outcomes should be extended to different conditions with caution. In perspective, a further investigation might examine the effects of the handgrip of the prime movers’ centroid placement.

## Conclusions

In conclusion, the front-LPD exhibited overall greater prime movers’ excitation compared with the back-LPD, also considering that the greater absolute external load and displacement may have affected the comparison. However, and more importantly, the front- and the back-LPD showed unique spatial excitation of the prime movers, depending on the unique characteristics of each variation.

Some practical considerations are needed. First of all, a quantitative comparison of the front- and the back-LPD would suggest that the former elicits more muscle excitation matched for relative external loads, thus appearing preferable. However, a more profound examination possible due to high-density EMG reveals that the primary muscles involved are stimulated differently. On this basis, both exercises should be included in practice. A further point regards the extensibility of both variations to all populations. As for the considerations previously made for the back-overhead press (Coratella et al., 2022b), the back-LPD requires greater gleno-humeral joint mobility to be performed safely and effectively. Indeed, people with poor mobility would be forced to flex the neck to let the bar pass backward. Additionally, a previous study reported that incorporating exercises passing from the “high-five” position may result in greater shoulder instability (Kolber et al., 2013). However, the same authors acknowledged possible confounding results from false positive evaluations, thus that the relationship may not be so clear (Kolber et al., 2013). Consequently, rather than excluding a priori the backward variation, one may accustom individuals to first tolerate and then safely and effectively perform such an initially less comfortable position. This would lead to a more posterior position, possibly decreasing kyphotic postures especially in sedentary people. Eventually, both the front- and the back-LPD should be prescribed to vary the spatial stimuli to the prime movers.

## References

[ref1] Andersen, V., Fimland, M. S., Mo, D.-A., Iversen, V. M., Larsen, T. M., Solheim, F., & Saeterbakken, A. H. (2019). Electromyographic comparison of the barbell deadlift using constant versus variable resistance in healthy, trained men. PloS One, 14(1), e0211021. 10.1371/journal.pone.0211021 30668589 PMC6342300

[ref2] Andersen, V., Fimland, M. S., Wiik, E., Skoglund, A., & Saeterbakken, A. H. (2014). Effects of Grip Width on Muscle Strength and Activation in the Lat Pull-Down: Journal of Strength and Conditioning Research, 28(4), 1135–1142. 10.1097/JSC.0000000000000232 24662157

[ref3] Barbero, M., Merletti, R., & Rainoldi, A. (2012). Atlas of Muscle Innervation Zones. Springer Milan. 10.1007/978-88-470-2463-2

[ref4] Besomi, M., Hodges, P. W., Clancy, E. A., Van Dieën, J., Hug, F., Lowery, M., Merletti, R., Søgaard, K., Wrigley, T., Besier, T., Carson, R. G., Disselhorst-Klug, C., Enoka, R. M., Falla, D., Farina, D., Gandevia, S., Holobar, A., Kiernan, M. C., McGill, K., … Tucker, K. (2020). Consensus for experimental design in electromyography (CEDE) project: Amplitude normalization matrix. Journal of Electromyography and Kinesiology, 53, 102438. 10.1016/j.jelekin.2020.102438 32569878

[ref5] Besomi, M., Hodges, P. W., Van Dieën, J., Carson, R. G., Clancy, E. A., Disselhorst-Klug, C., Holobar, A., Hug, F., Kiernan, M. C., Lowery, M., McGill, K., Merletti, R., Perreault, E., Søgaard, K., Tucker, K., Besier, T., Enoka, R., Falla, D., Farina, D., … Wrigley, T. (2019). Consensus for experimental design in electromyography (CEDE) project: Electrode selection matrix. Journal of Electromyography and Kinesiology: Official Journal of the International Society of Electrophysiological Kinesiology, 48, 128–144. 10.1016/j.jelekin.2019.07.008 31352156

[ref6] Błażkiewicz, M., & Hadamus, A. (2022). The Effect of the Weight and Type of Equipment on Shoulder and Back Muscle Activity in Surface Electromyography during the Overhead Press-Preliminary Report. Sensors (Basel, Switzerland), 22(24), 9762. 10.3390/s22249762 36560129 PMC9781216

[ref7] Cabral, H. V., de Souza, L. M. L., de Oliveira, L. F., & Vieira, T. M. (2022). Non-uniform excitation of the pectoralis major muscle during flat and inclined bench press exercises. Scandinavian Journal of Medicine & Science in Sports, 32(2), 381–390. 10.1111/sms.14082 34644424

[ref8] Campanini, I., Merlo, A., Disselhorst-Klug, C., Mesin, L., Muceli, S., & Merletti, R. (2022). Fundamental Concepts of Bipolar and High-Density Surface EMG Understanding and Teaching for Clinical, Occupational, and Sport Applications: Origin, Detection, and Main Errors. Sensors (Basel, Switzerland), 22(11), 4150. 10.3390/s22114150 35684769 PMC9185290

[ref9] Clark, D. R., Lambert, M. I., & Hunter, A. M. (2012). Muscle Activation in the Loaded Free Barbell Squat: A Brief Review. The Journal of Strength & Conditioning Research, 26(4), 1169. 10.1519/JSC.0b013e31822d533d 22373894

[ref10] Cohen, J. (1988). Statistical power analysis for the behavioral sciences (2nd ed). L. Erlbaum Associates.

[ref11] Coratella, G. (2022). Appropriate Reporting of Exercise Variables in Resistance Training Protocols: Much more than Load and Number of Repetitions. Sports Medicine-Open, 8, 99. 10.1186/s40798-022-00492-1 35907047 PMC9339067

[ref12] Coratella, G., Beato, M., Bertinato, L., Milanese, C., Venturelli, M., & Schena, F. (2022). Including the Eccentric Phase in Resistance Training to Counteract the Effects of Detraining in Women: A Randomized Controlled Trial. Journal of Strength and Conditioning Research, 36(11), 3023–3031. 10.1519/JSC.0000000000004039 34537804 PMC10842669

[ref13] Coratella, G., & Bertinato, L. (2015). Isoload vs isokinetic eccentric exercise: A direct comparison of exercise-induced muscle damage and repeated bout effect. Sport Sciences for Health, 11(1), 87–96. 10.1007/s11332-014-0213-x

[ref14] Coratella, G., Galas, A., Campa, F., Pedrinolla, A., Schena, F., & Venturelli, M. (2022). The Eccentric Phase in Unilateral Resistance Training Enhances and Preserves the Contralateral Knee Extensors Strength Gains After Detraining in Women: A Randomized Controlled Trial. Frontiers in Physiology, 13, 788473. 10.3389/fphys.2022.788473 35309062 PMC8928196

[ref15] Coratella, G., Tornatore, G., Caccavale, F., Longo, S., Esposito, F., & Cè, E. (2021). The Activation of Gluteal, Thigh, and Lower Back Muscles in Different Squat Variations Performed by Competitive Bodybuilders: Implications for Resistance Training. International Journal of Environmental Research and Public Health, 18(2), 772. 10.3390/ijerph18020772 33477561 PMC7831128

[ref16] Coratella, G., Tornatore, G., Longo, S., Esposito, F., & Cè, E. (2020a). An Electromyographic Analysis of Lateral Raise Variations and Frontal Raise in Competitive Bodybuilders. International Journal of Environmental Research and Public Health, 17(17), 6015. 10.3390/ijerph17176015 32824894 PMC7503819

[ref17] Coratella, G., Tornatore, G., Longo, S., Esposito, F., & Cè, E. (2020b). Specific prime movers’ excitation during free-weight bench press variations and chest press machine in competitive bodybuilders. European Journal of Sport Science, 20(5), 571–579. 10.1080/17461391.2019.1655101 31397215

[ref18] Coratella, G., Tornatore, G., Longo, S., Esposito, F., & Cè, E. (2022a). An Electromyographic Analysis of Romanian, Step-Romanian, and Stiff-Leg Deadlift: Implication for Resistance Training. International Journal of Environmental Research and Public Health, 19(3), 1903. 10.3390/ijerph19031903 35162922 PMC8835508

[ref19] Coratella, G., Tornatore, G., Longo, S., Esposito, F., & Cè, E. (2022b). Front vs Back and Barbell vs Machine Overhead Press: An Electromyographic Analysis and Implications For Resistance Training. Frontiers in Physiology, 13, 825880. 10.3389/fphys.2022.825880 35936912 PMC9354811

[ref20] Coratella, G., Tornatore, G., Longo, S., Esposito, F., & Cè, E. (2023). Bilateral Biceps Curl Shows Distinct Biceps Brachii and Anterior Deltoid Excitation Comparing Straight vs. EZ Barbell Coupled with Arms Flexion/No-Flexion. Journal of Functional Morphology and Kinesiology, 8(1), Articolo 1. 10.3390/jfmk8010013 PMC9944112 36810497

[ref21] Coratella, G., Tornatore, G., Longo, S., Toninelli, N., Padovan, R., Esposito, F., & Cè, E. (2023). Biceps Brachii and Brachioradialis Excitation in Biceps Curl Exercise: Different Handgrips, Different Synergy. Sports, 11(3), Articolo 3. 10.3390/sports11030064 PMC10054060 36976950

[ref22] Duchateau, J., & Enoka, R. M. (2016). Neural control of lengthening contractions. The Journal of Experimental Biology, 219(Pt 2), 197–204. 10.1242/jeb.123158 26792331

[ref23] Frost, D. M., Cronin, J. B., & Newton, R. U. (2008). A comparison of the kinematics, kinetics and muscle activity between pneumatic and free weight resistance. European Journal of Applied Physiology, 104(6), 937–956. 10.1007/s00421-008-0821-8 18830619

[ref24] Fujita, R. A., Silva, N. R. S., Bedo, B. L. S., Santiago, P. R. P., Gentil, P. R. V., & Gomes, M. M. (2020). Mind–Muscle Connection: Limited Effect of Verbal Instructions on Muscle Activity in a Seated Row Exercise. Perceptual and Motor Skills, 127(5), 925–938. 10.1177/0031512520926369 32448047

[ref25] Gallina, A., Merletti, R., & Gazzoni, M. (2013). Uneven spatial distribution of surface EMG: What does it mean? European Journal of Applied Physiology, 113(4), 887–894. 10.1007/s00421-012-2498-2 23001682

[ref26] Hegyi, A., Csala, D., Péter, A., Finni, T., & Cronin, N. J. (2019). High-density electromyography activity in various hamstring exercises. Scandinavian Journal of Medicine & Science in Sports, 29(1), 34–43. 10.1111/sms.13303 30230042

[ref27] Hopkins, W. G., Marshall, S. W., Batterham, A. M., & Hanin, J. (2009). Progressive Statistics for Studies in Sports Medicine and Exercise Science. Medicine & Science in Sports & Exercise, 41(1), 3. 10.1249/MSS.0b013e31818cb278 19092709

[ref28] Jaworski, J., Ambroży, T., Lech, G., Spieszny, M., Bujas, P., Żak, M., & Chwała, W. (2020). Absolute and Relative Reliability of Several Measures of Static Postural Stability Calculated Using a GYKO Inertial Sensor System. Acta of Bioengineering & Biomechanics, 22(2), 1–14. 10.37190/abb-01502-2019-02 32868935

[ref29] Kolber, M. J., Corrao, M., & Hanney, W. J. (2013). Characteristics of anterior shoulder instability and hyperlaxity in the weight-training population. Journal of Strength and Conditioning Research, 27(5), 1333–1339. 10.1519/JSC.0b013e318269f776 22836608

[ref30] Kompf, J., & Arandjelović, O. (2016). Understanding and Overcoming the Sticking Point in Resistance Exercise. Sports Medicine (Auckland, N.Z.), 46(6), 751–762. 10.1007/s40279-015-0460-2 26758462 PMC4887540

[ref31] Marcolin, G., Panizzolo, F. A., Petrone, N., Moro, T., Grigoletto, D., Piccolo, D., & Paoli, A. (2018). Differences in electromyographic activity of biceps brachii and brachioradialis while performing three variants of curl. PeerJ, 6, e5165. 10.7717/peerj.5165 30013836 PMC6047503

[ref32] Marri, K., & Swaminathan, R. (2016). Analyzing the influence of curl speed in fatiguing biceps brachii muscles using sEMG signals and multifractal detrended moving average algorithm. *2016 38th Annual International Conference of the IEEE Engineering in Medicine and Biology Society (EMBC)*, 3658–3661. 10.1109/EMBC.2016.7591521 28269087

[ref33] Martín-Fuentes, I., Oliva-Lozano, J. M., & Muyor, J. M. (2020). Electromyographic activity in deadlift exercise and its variants. A systematic review. PloS One, 15(2), e0229507. 10.1371/journal.pone.0229507 32107499 PMC7046193

[ref34] Merletti, R., & Muceli, S. (2019). Tutorial. Surface EMG detection in space and time: Best practices. Journal of Electromyography and Kinesiology: Official Journal of the International Society of Electrophysiological Kinesiology, 49, 102363. 10.1016/j.jelekin.2019.102363 31665683

[ref35] Merletti, R., Rainoldi, A., & Farina, D. (2001a). Surface Electromyography for Noninvasive Characterization of Muscle. *Exercise and Sport Sciences Reviews*, 29(1), 20. 11210442 10.1097/00003677-200101000-00005

[ref36] Merletti, R., Rainoldi, A., & Farina, D. (2001b). Surface electromyography for noninvasive characterization of muscle. Exercise and Sport Sciences Reviews, 29(1), 20–25. 10.1097/00003677-200101000-00005 11210442

[ref37] Rawska, M., Gepfert, M., Mostowik, A., Krzysztofik, M., Wojdała, G., & Lulińska, A. et al. (2019). Does blood flow restriction influence the maximal number of repetitions performed during the bench press? A pilot study. Balt J Health Phys Activ, 11(4), 9-17. 10.29359/BJHPA.11.4.02

[ref38] Reinold, M. M., Macrina, L. C., Wilk, K. E., Fleisig, G. S., Dun, S., Barrentine, S. W., Ellerbusch, M. T., & Andrews, J. R. (2007). Electromyographic Analysis of the Supraspinatus and Deltoid Muscles During 3 Common Rehabilitation Exercises. Journal of Athletic Training, 42(4), 464–469. 18174934 PMC2140071

[ref39] Rodriguez-Falces, J., Negro, F., Gonzalez-Izal, M., & Farina, D. (2013). Spatial distribution of surface action potentials generated by individual motor units in the human biceps brachii muscle. Journal of Electromyography and Kinesiology, 23(4), 766–777. 10.1016/j.jelekin.2013.03.011 23619102

[ref40] Saeterbakken, A. H., Stien, N., Pedersen, H., Solstad, T. E. J., Cumming, K. T., & Andersen, V. (2021). The Effect of Grip Width on Muscle Strength and Electromyographic Activity in Bench Press among Novice- and Resistance-Trained Men. International Journal of Environmental Research and Public Health, 18(12), 6444. 10.3390/ijerph18126444 34198674 PMC8296276

[ref41] Signorile, J. F., Zink, A. J., & Szwed, S. P. (2002). A comparative electromyographical investigation of muscle utilization patterns using various hand positions during the lat pull-down. Journal of Strength and Conditioning Research, 16(4), 539–546. 12423182

[ref42] Sperandei, S., Barros, M. A. P., Silveira-Júnior, P. C. S., & Oliveira, C. G. (2009). Electromyographic Analysis of Three Different Types of Lat Pull-Down. Journal of Strength and Conditioning Research, 23(7), 2033–2038. 10.1519/JSC.0b013e3181b8d30a 19855327

[ref43] Stastny, P., Gołaś, A., Blazek, D., Maszczyk, A., Wilk, M., Pietraszewski, P., Petr, M., Uhlir, P., and Zając, A. (2017). A systematic review of surface electromyography analyses of the bench press movement task. PLoS ONE, 12(2), e0171632. 10.1371/journal.pone.0171632 28170449 PMC5295722

[ref44] Stronska, K., Bojacz, P., Golas, A., Maszczyk, A., Zajac, A., & Stastny, P. (2018). Muscle activity during the incline shoulder press in relation to the exercise intensity. Acta Gymnica, 48(4), 141–146. 10.5507/ag.2018.019

[ref45] Strońska, K., Trebert, M., Gołaś, A., Maszczyk, A., & Zając, A. (2018). Changes in EMG activity of the prime movers during 10 sets of the flat bench press performed to concentric failure. Balt J Health Phys Activ, 10, 22-29. 10.29359/BJHPA.10.1.02

[ref46] Stronska, K., Golas, A., Wilk, M., Zajac, A., Maszczyk, A., & Stastny, P. (2022). The effect of targeted resistance training on bench press performance and the alternation of prime mover muscle activation patterns. Sports Biomechanics, 21(10), 1262–1276. 10.1080/14763141.2020.1752790 32460639

[ref47] Tsoukos, A., and Bogdanis, G. C. (2023). Lower Fatigue in the Eccentric than the Concentric Phase of a Bench Press Set Executed with Maximum Velocity to Failure Against Both Heavy and Light Loads. Journal of Human Kinetics, 87, 119–129. 10.5114/jhk/168792 PMC10407316 37559769

[ref48] van den Tillaar, R., Andersen, V., & Saeterbakken, A. H. (2019). Comparison of muscle activation and kinematics during free-weight back squats with different loads. PloS One, 14(5), e0217044. 10.1371/journal.pone.0217044 31095625 PMC6521994

[ref49] Vieira, T. M., & Botter, A. (2021). The Accurate Assessment of Muscle Excitation Requires the Detection of Multiple Surface Electromyograms. Exercise and Sport Sciences Reviews, 49(1), 23–34. 10.1249/JES.0000000000000240 33044329

[ref50] Watanabe, K., Kouzaki, M., & Moritani, T. (2012). Task-dependent spatial distribution of neural activation pattern in human rectus femoris muscle. Journal of Electromyography and Kinesiology, 22(2), 251–258. 10.1016/j.jelekin.2011.11.004 22153052

[ref51] Wojdala, G., Trybulski, R., Bichowska, M., and Krzysztofik, M. (2022). A Comparison of Electromyographic Inter-Limb Asymmetry During a Standard Versus a Sling Shot Assisted Bench Press Exercise. Journal of Human Kinetics, 83, 223–234. 10.2478/hukin-2022-0084 36157940 PMC9465753

